# Retraction Note: BET inhibitor I-BET151 sensitizes GBM cells to temozolomide via PUMA induction

**DOI:** 10.1038/s41417-022-00536-4

**Published:** 2022-09-20

**Authors:** Zhicheng Yao, Shida Yang, Hongyou Zhao, Huike Yang, Xin Jiang

**Affiliations:** 1 Department of Neurology, The People’s Hospital of Liaoning Province, Shenyang, Liaoning China; 2 Department of Laboratory Medicine, The People’s Hospital of Liaoning Province, Shenyang, Liaoning China; 3 Department of Genetics, Louisiana state university health science center, New Orleans, LA USA; 4 Department of Anatomy, Harbin Medical University, Harbin, Heilongjiang China

Retraction of: *Cancer Gene Therapy* 10.1038/s41417-018-0068-4, published online 01 February 2019

The Editor-in-Chief has retracted this article at the authors’ request. After publication, the authors noticed that incorrect images were used in Fig. 2E to inform their conclusions. The authors therefore no longer have confidence in the presented results and data reproducibility.

ZY and XJ agree to this retraction. SY and HY have not responded to any correspondence from the editor or publisher about this retraction. The Editor has not been able to obtain a current email address for HZ.

